# High Burden of COVID-19-Associated Pulmonary Aspergillosis in Severely Immunocompromised Patients Requiring Mechanical Ventilation

**DOI:** 10.1093/cid/ciad546

**Published:** 2023-09-11

**Authors:** Simon Feys, Katrien Lagrou, Hanne Moon Lauwers, Koen Haenen, Cato Jacobs, Marius Brusselmans, Yves Debaveye, Greet Hermans, Martin Hoenigl, Johan Maertens, Philippe Meersseman, Marijke Peetermans, Isabel Spriet, Christophe Vandenbriele, Lore Vanderbeke, Robin Vos, Eric Van Wijngaerden, Alexander Wilmer, Joost Wauters

**Affiliations:** Medical Intensive Care Unit, University Hospitals Leuven, Leuven, Belgium; Department of Microbiology, Immunology and Transplantation, KU Leuven, Leuven, Belgium; Department of Microbiology, Immunology and Transplantation, KU Leuven, Leuven, Belgium; Department of Laboratory Medicine and National Reference Center for Mycosis, University Hospitals Leuven, Leuven, Belgium; Medical Intensive Care Unit, University Hospitals Leuven, Leuven, Belgium; Medical Intensive Care Unit, University Hospitals Leuven, Leuven, Belgium; Medical Intensive Care Unit, University Hospitals Leuven, Leuven, Belgium; Leuven Biostatistics and Statistical Bioinformatics Center (L-BioStat), KU Leuven, Leuven, Belgium; Department of Intensive Care Medicine, University Hospitals Leuven, Leuven, Belgium; Department of Cellular and Molecular Medicine, KU Leuven, Leuven, Belgium; Medical Intensive Care Unit, University Hospitals Leuven, Leuven, Belgium; Department of Cellular and Molecular Medicine, KU Leuven, Leuven, Belgium; Division of Infectious Diseases, ECMM Excellence Center, Department of Internal Medicine, Medical University of Graz, Graz, Austria; Bio TechMed, Graz, Austria; Translational Medical Mycology Research Group, Medical University of Graz, Graz, Austria; Department of Microbiology, Immunology and Transplantation, KU Leuven, Leuven, Belgium; Department of Hematology, University Hospitals Leuven, Leuven, Belgium; Medical Intensive Care Unit, University Hospitals Leuven, Leuven, Belgium; Department of Microbiology, Immunology and Transplantation, KU Leuven, Leuven, Belgium; Medical Intensive Care Unit, University Hospitals Leuven, Leuven, Belgium; Department of Microbiology, Immunology and Transplantation, KU Leuven, Leuven, Belgium; Pharmacy Department, University Hospitals Leuven, Leuven, Belgium; Department of Pharmaceutical and Pharmacological Sciences, KU Leuven, Leuven, Belgium; Department of Cardiovascular Diseases, University Hospitals Leuven, Leuven, Belgium; Department of Cardiovascular Sciences, KU Leuven, Leuven, Belgium; Department of General Internal Medicine, University Hospitals Leuven, Leuven, Belgium; Department of Respiratory Diseases, University Hospitals Leuven, Leuven, Belgium; Department of Chronic Diseases and Metabolism, KU Leuven, Leuven, Belgium; Department of Microbiology, Immunology and Transplantation, KU Leuven, Leuven, Belgium; Department of General Internal Medicine, University Hospitals Leuven, Leuven, Belgium; Medical Intensive Care Unit, University Hospitals Leuven, Leuven, Belgium; Department of Microbiology, Immunology and Transplantation, KU Leuven, Leuven, Belgium; Medical Intensive Care Unit, University Hospitals Leuven, Leuven, Belgium; Department of Microbiology, Immunology and Transplantation, KU Leuven, Leuven, Belgium

**Keywords:** CAPA, COVID-19, aspergillosis, immunocompromised, critical care

## Abstract

**Background:**

Coronavirus disease 2019 (COVID-19)–associated pulmonary aspergillosis (CAPA) is a frequent superinfection in critically ill patients with COVID-19 and is associated with increased mortality rates. The increasing proportion of severely immunocompromised patients with COVID-19 who require mechanical ventilation warrants research into the incidence and impact of CAPA during the vaccination era.

**Methods:**

We performed a retrospective, monocentric, observational study. We collected data from adult patients with severe COVID-19 requiring mechanical ventilation who were admitted to the intensive care unit (ICU) of University Hospitals Leuven, a tertiary referral center, between 1 March 2020 and 14 November 2022. Probable or proven CAPA was diagnosed according to the 2020 European Confederation for Medical Mycology/International Society for Human and Animal Mycology (ECMM/ISHAM) criteria.

**Results:**

We included 335 patients. Bronchoalveolar lavage sampling was performed in 300 (90%), and CAPA was diagnosed in 112 (33%). The incidence of CAPA was 62% (50 of 81 patients) in European Organisation for Research and Treatment of Cancer (EORTC)/Mycosis Study Group Education and Research Consortium (MSGERC) host factor–positive patients, compared with 24% (62 of 254) in host factor–negative patients. The incidence of CAPA was significantly higher in the vaccination era, increasing from 24% (57 of 241) in patients admitted to the ICU before October 2021 to 59% (55 of 94) in those admitted since then. Both EORTC/MSGERC host factors and ICU admission in the vaccination era were independently associated with CAPA development. CAPA remained an independent risk factor associated with mortality risk during the vaccination era.

**Conclusions:**

The presence of EORTC/MSGERC host factors for invasive mold disease is associated with increased CAPA incidence and worse outcome parameters, and it is the main driver for the significantly higher incidence of CAPA in the vaccination era. Our findings warrant investigation of antifungal prophylaxis in critically ill patients with COVID-19.

## Teaser

The incidence of coronavirus disease 2019 (COVID-19)–associated pulmonary aspergillosis (CAPA) increased significantly in mechanically ventilated patients with COVID-19 as the proportion of severely immunocompromised patients increased since widespread vaccination. CAPA stays independently associated with mortality rate in the vaccination era.

## Graphical Abstract

**Graphical Abstract:**
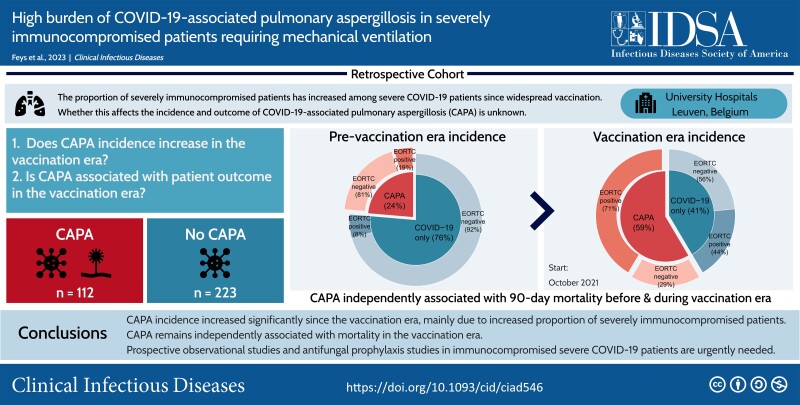
This graphical abstract is also available at Tidbit: https://tidbitapp.io/tidbits/high-burden-of-covid-19-associated-pulmonary-aspergillosis-capa-in-severelyimmunocompromised-patients-requiring-mechanical-ventilation

The rapid development and widespread administration of highly effective vaccines against severe acute respiratory syndrome coronavirus 2 (SARS-CoV-2) are among the greatest medical achievements of the past decades. Moreover, optimization of coronavirus disease 2019 (COVID-19) management, the development of population immunity and the circulation of the generally less virulent Omicron variants have curbed the overall severity of this viral infection. Indeed, the general COVID-19 mortality rate has decreased significantly since the start of the pandemic [1].

However, in 2022 COVID-19 still caused on average >10 000 deaths worldwide per week [1]. Moreover, a recent study found that COVID-19 caused by the currently circulating Omicron subvariants is still deadlier than seasonal influenza [2]. The vulnerable population now mainly consists of immunocompromised individuals, who are inherently prone to more severe disease and who may encounter breakthrough infection despite vaccination [3–6].

COVID-19–associated pulmonary aspergillosis (CAPA) is an important possible complication in patients who require mechanical ventilation for severe COVID-19 [7]. This superinfection caused by the fungus *Aspergillus* is frequent and occurs in approximately 10%–15% of patients included in studies with a thorough fungal workup [8–10]. Moreover, it is associated with an increased mortality rate [8–10].

Nevertheless, several knowledge gaps persist concerning CAPA. Despite numerous observational studies published to date, data on the incidence of CAPA in the vaccination era are lacking. Given the shifted patient profile, the prevalence of classic immunity-related host factors that predispose to the development of invasive mold disease has likely increased among patients with severe COVID-19. These factors have been defined by the European Organisation for Research and Treatment of Cancer (EORTC) and the Mycosis Study Group Education and Research Consortium (MSGERC) outside the context of severe viral disease [11]. While it has become clear that severe COVID-19 on its own is a risk factor for invasive aspergillosis through several pathophysiological mechanisms [12–14], only a few studies have looked specifically at the impact of EORTC/MSGERC host factors in the context of severe COVID-19, with conflicting results [10, 15–17]. In the current study, we investigated the impact of the vaccination era and of EORTC/MSGERC host factors on the incidence and outcome characteristics of CAPA in patients with severe COVID-19.

## METHODS

We conducted a retrospective observational study of patients with COVID-19 admitted to the intensive care units (ICUs) of University Hospitals Leuven, a tertiary referral center, between 1 March 2020 and 14 November 2022. Inclusion criteria were presence of a positive PCR result for SARS-CoV-2 within 14 days before or 10 days after ICU admission, respiratory distress as the main reason for ICU admission, a requirement for ≥24 hours of mechanical ventilation because of COVID-19 during the ICU stay, and age ≥18 years. Exclusion criteria were a recent history of invasive aspergillosis (within 3 months before ICU admission) and/or active treatment for invasive aspergillosis at the time of ICU admission. Diagnosis of CAPA was based on the European Confederation for Medical Mycology/International Society for Human and Animal Mycology (ECMM/ISHAM) criteria in combination with clinical suspicion for CAPA [18]. We considered only probable or proven CAPA (according to the ECMM/ISHAM criteria) as CAPA positive in this study. The study protocol was approved and the need for informed consent waived by the Ethical Committee of University Hospitals Leuven (as part of protocol S65588).

To compare the prevaccination and the vaccination era, we divided the patients into 2 groups: those admitted to the ICU before and those admitted since 4 October 2021 (referred to as hereafter as “before October 2021” or “since October 2021”). The cutoff date of 4 October 2021 marks the start of the fourth COVID-19 wave in Belgium [19]. Importantly, this is seen as the first wave in Belgium with a high vaccination prevalence, as the percentage of people who completed vaccination reached 70% in October 2021 [20]. In this month, the Flemish region, in which University Hospitals Leuven operates, reached a completed vaccination rate of 80%. Completed vaccination was defined as having received 2 COVID-19 vaccination doses of messenger RNA (mRNA)–based BNT162b2 (Comirnaty) or mRNA-1273 (Spikevax), 2 doses of viral vector-based AZD1222 (Vaxzevria), or 1 dose of viral vector-based Ad26.COV2.S (Jcovden) before hospitalization. We opted to divide the groups according to ICU admission date rather than individual vaccination status, as the latter would neglect the impact of population immunity.

For univariable analyses, we compared categorical variables with the Fisher exact test and continuous variables with Student *t* or Mann-Whitney *U* tests where appropriate, and we performed time-to-event analyses using log-rank tests (for landmark 90-day mortality rate analyses) and Cox regression analysis (for 90-day mortality analysis with CAPA as a time-dependent variable). For multivariable analyses, we performed a binary logistic regression to investigate relevant risk factors for CAPA development, including (among others) EORTC/MSGERC host factor status and ICU admittance before or during the vaccination era. In addition, a Fine and Gray subdistribution hazard model was used to assess the impact of EORTC/MSGERC host factors and the influence of the vaccination era on CAPA incidence, correcting for competing risks (weaning from mechanical ventilation or death). We performed multivariable Cox regression analysis with CAPA as a time-dependent variable to investigate the association of risk factors with survival in all patients and in patients admitted to ICU since the vaccination era specifically. We used a 2-sided alternative hypothesis at the 5% significance level for all statistical analyses. We did not perform statistical corrections for multiple testing. All statistical analyses were performed using R software (version 4.2.1).

## RESULTS

### Overall CAPA Characteristics

Between 1 March 2020 and 14 November 2022, 836 patients with severe COVID-19 were admitted to the ICUs of our center, of whom 335 mechanically ventilated patients were eligible for inclusion in the study (Supplementary Figure 1). The main clinical characteristics are provided in Table 1.

**Table 1.** **Clinical Characteristics of Patients With or Without Coronavirus Disease**–**Associated Pulmonary Aspergillosis**

Characteristic	Patients, No. (%)^a^			*P* Value^b^
	All Patients (N = 335)	CAPA (n = 112)	Non-CAPA (n = 223)	
Male sex	238 (71)	86 (77)	152 (68)	.13
Age, mean (SD), y	63 (11)	65 (9.8)	62 (12)	.006^c^
BMI, median (IQR)^d^	28 (25–33)	27 (25–31)	29 (25–33)	.053
COPD	42 (13)	18 (16)	24 (11)	.17
Liver cirrhosis	3 (0.90)	1 (0.89)	2 (0.90)	>.99
Diabetes mellitus	122 (36)	35 (31)	87 (39)	.19
EORTC/MSGERC host factor^e^	81 (24)	50 (45)	31 (14)	<.001^c^
Solid organ transplant	50 (15)	32 (29)	18 (8.1)	<.001^c^
Lung transplant	29 (8.7)	18 (16)	11 (4.9)	.002^c^
Kidney transplant	14 (4.2)	10 (8.9)	4 (1.8)	.004^c^
Liver transplant	3 (0.90)	2 (1.8)	1 (0.45)	.26
Heart transplant	5 (1.5)	3 (2.7)	2 (0.90)	.34
Hematological cancer	21 (6.3)	13 (12)	8 (3.6)	.007^c^
Allogeneic HSCT	5 (1.5)	3 (2.7)	2 (0.90)	.34
Acute GVHD (grade III–IV) involving gut, lungs, or liver refractory to first-line CS	2 (0.60)	1 (0.89)	1 (0.45)	>.99
Recent prolonged neutropenia	1 (0.30)	0 (0)	1 (0.45)	>.99
Recent prolonged high-dose CS	6 (1.8)	3 (2.7)	3 (1.3)	.41
T- or B-cell immunosuppressants	71 (21)	44 (39)	27 (12)	<.001^c^
Inherited severe immunodeficiency	0 (0)	0 (0)	0 (0)	>.99
Low-dose CS as long-term medication^f^	64 (19)	38 (34)	26 (12)	<.001^c^
APACHE II score at ICU admission, median (IQR)	19 (16–26) (n = 280)	21 (17–25) (n = 98)	18 (15–26) (n = 182)	.19
Charlson comorbidity index at ICU admission, median (IQR)	3 (2–4)	4 (3–5)	3 (2–4)	<.001^c^
CS (daily dose, ≥20 mg prednisone equivalent) as treatment for COVID-19 during ICU stay	306 (91)	109 (97)	197 (88)	.006^c^
Tocilizumab during hospital stay	16 (4.8)	2 (1.8)	14 (6.3)	.10
Received MV	335 (100)	112 (100)	223 (100)	>.99
Duration of MV, median (IQR), d	14 (8–25)	19 (11–32)	13 (6–21)	<.001^c^
Received ECMO	68 (20)	17 (15)	51 (23)	.11
Duration of ECMO, median (IQR), d	14 (9–24) n = 68	18 (13–28) n = 17	13 (9–22) n = 51	.13
Required renal replacement therapy	75 (22)	37 (33)	38 (17)	.001^c^
Duration of ICU stay, median (IQR), d	24 (15–40)	32 (21–49)	21 (13–33)	<.001^c^
Duration of hospital stay, median (IQR), d	38 (24–60)	46 (30–79)	33 (23–53)	<.001^c^
Death within 90 d after ICU admission	101 (30)	54 (48)	47 (21)	<.001
≥1 BAL sample obtained	300 (90)	112 (100)	188 (84)	<.001^c^
≥1 BAL GM test	290 (87)	111 (99)	179 (80)	<.001^c^
≥1 Serum GM test	188 (56)	85 (76)	103 (43)	<.001^c^

Abbreviations: APACHE II, Acute Physiology and Chronic Health Evaluation II; BAL, bronchoalveolar lavage; BMI, body mass index; CAPA, COVID-19–associated pulmonary aspergillosis; COPD, chronic obstructive pulmonary disease; COVID-19, coronavirus disease 2019; CS, corticosteroid; ECMO, extracorporeal membrane oxygenation; EORTC, European Organisation for Research and Treatment of Cancer; GM, galactomannan; GVHD, graft-vs-host disease; HSCT, hematopoietic stem cell transplant; ICU, intensive care unit; IQR, interquartile range; MV, mechanical ventilation; MSGERC, Mycosis Study Group Education and Research Consortium; SD, standard deviation.

^a^Data represent no. (%) of patients unless otherwise specified.

^b^ *P* values calculated for categorical variables with Fisher exact test and for continuous variables with Student *t* or Mann-Whitney *U* test where appropriate.

^c^Significant at *P* < .05.

^d^BMI calculated as weight in kilograms divided by height in meters squared.

^e^EORTC/MSGERC host factors for invasive mold disease [11].

^f^Daily dose below the EORTC/MSGERC CS host factor cutoff (≥0.3 mg/kg/d for ≥3 weeks during the past 60 days) as long-term home medication.

**Figure 1.**
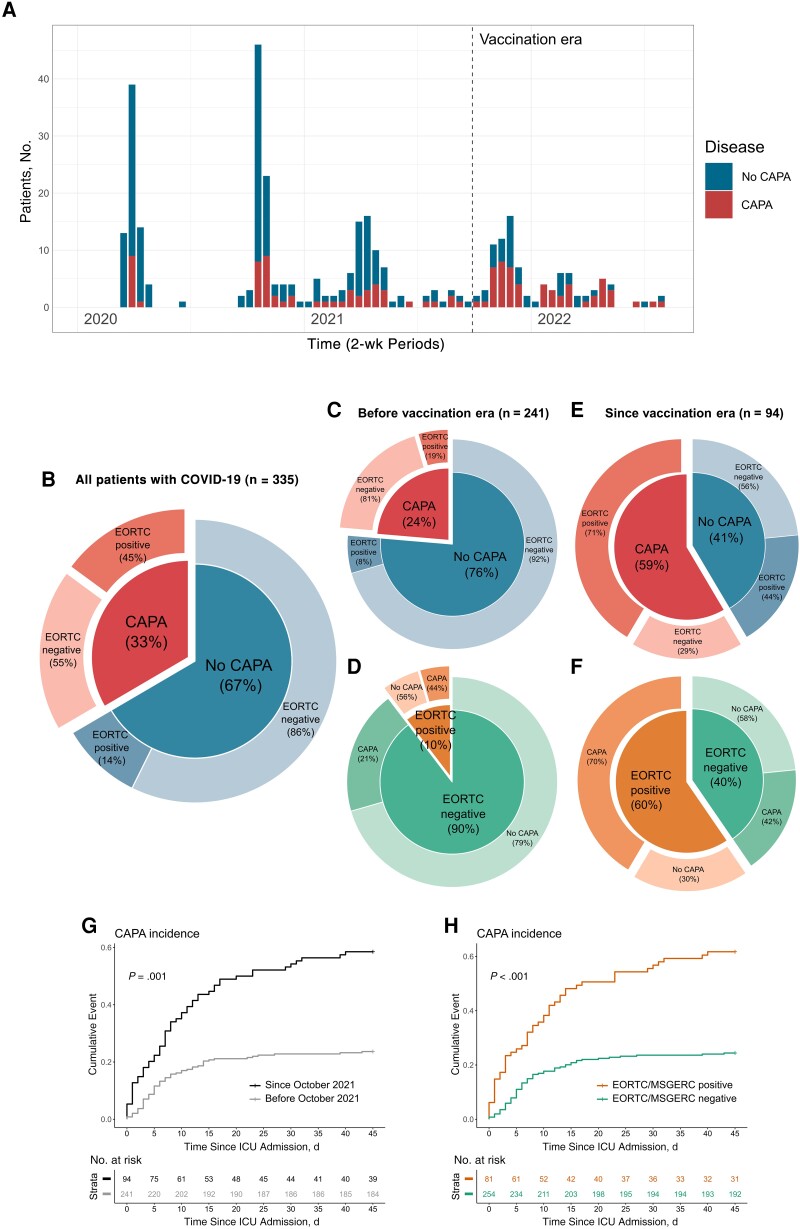
Incidence of coronavirus disease 2019 (COVID-19)–associated pulmonary aspergillosis (CAPA) as a function of the vaccination era and European Organisation for Research and Treatment of Cancer (EORTC)/Mycosis Study Group Education and Research Consortium (MSGERC) host factors. *A*, Incidence of CAPA displayed by 2-week periods within the time frame of inclusion for this study. *B*–*F*, Incidence of CAPA and prevalence of EORTC/MSGERC (shortened to EORTC in *B–F*) host factors in all patients (*B*) and in patients, subdivided into those admitted to the intensive care unit (ICU) before (*C, D*) or since (*E, F*) the beginning of vaccination era (October 2021). *G, H*, Cumulative incidence curves depicting the 45-day incidence of CAPA. *G*, Stratification according to admittance before or since the vaccination era (October 2021). *H*, Stratification according to presence of the EORTC/MSGERC host factor for invasive mold infection. *P* values were obtained using the Fine and Gray model (see Supplementary Figure 2).

Of the 335 patients, 112 (33%) received diagnoses of probable or proven CAPA during their ICU stay, according to the ECMM/ISHAM criteria (Figure 1) [18]. Importantly, 90% of patients in the COVID-19 cohort (300 of 335) provided ≥1 bronchoalveolar lavage (BAL) sample during the ICU stay, and BAL galactomannan (GM) testing was performed in 290 (87%) of the patients.

Patients with CAPA were slightly older than those without CAPA (mean age, 65 vs 62 years, respectively; *P* = .006). EORTC/MSGERC host factors for invasive mold disease were present in 50 (45%) of patients with CAPA versus 31 (14%) of patients without CAPA (*P* < .001), and they mainly consisted of the presence of a solid organ transplant and/or use of T- or B-cell immunosuppressants. Use of low-dose corticosteroids as home medication was present in 38 (34%) of patients with CAPA versus 26 (12%) without CAPA (*P* < .0001). Other medical history components linked to development of invasive aspergillosis, such as chronic obstructive pulmonary disease or liver cirrhosis, did not differ significantly between patients with or without CAPA. Patients with CAPA had a significantly higher Charlson comorbidity index at ICU admission than patients without CAPA (median [interquartile range (IQR)], 4 [3–5] vs 3 [2–4]; *P* < .001), while we found no significant difference in Acute Physiology and Chronic Health Evaluation II (APACHE II) scores. The overall number of patients who received corticosteroids during their ICU stay was high, with 109 (97%) of the patients with CAPA and 197 (88%) of those without CAPA having received a course of daily prednisone equivalent dose ≥20 mg as treatment for COVID-19.

**Figure 2.**
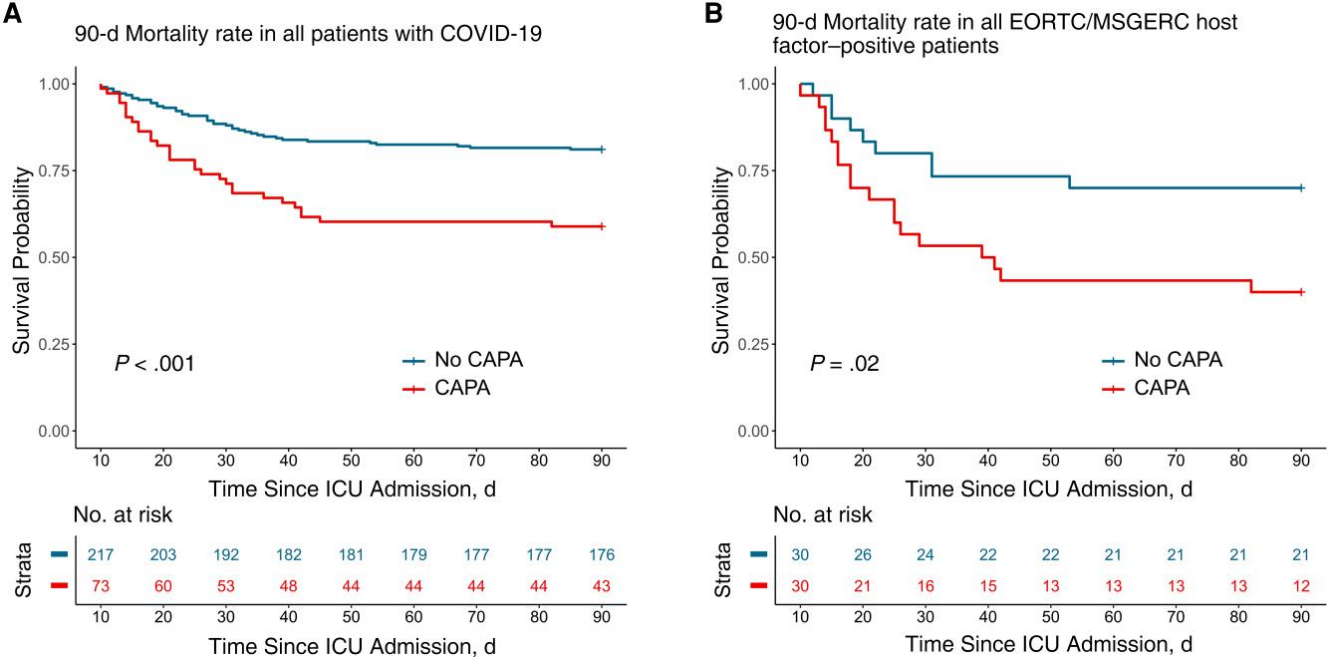
Association of coronavirus disease 2019 (COVID-19)–associated pulmonary aspergillosis (CAPA) with 90-day mortality rate. Kaplan-Meier curves depict the 90-day mortality rates in all patients (*A*) and in European Organisation for Research and Treatment of Cancer (EORTC)/Mycosis Study Group Education and Research Consortium (MSGERC) host factor-positive patients (*B*). Only patients who were still alive 10 days after intensive care unit (ICU) admission were included in this analysis. For the CAPA group, only patients whose CAPA developed within 10 days after ICU admission were included. Log-rank *P* values are shown.

**Figure 3.**
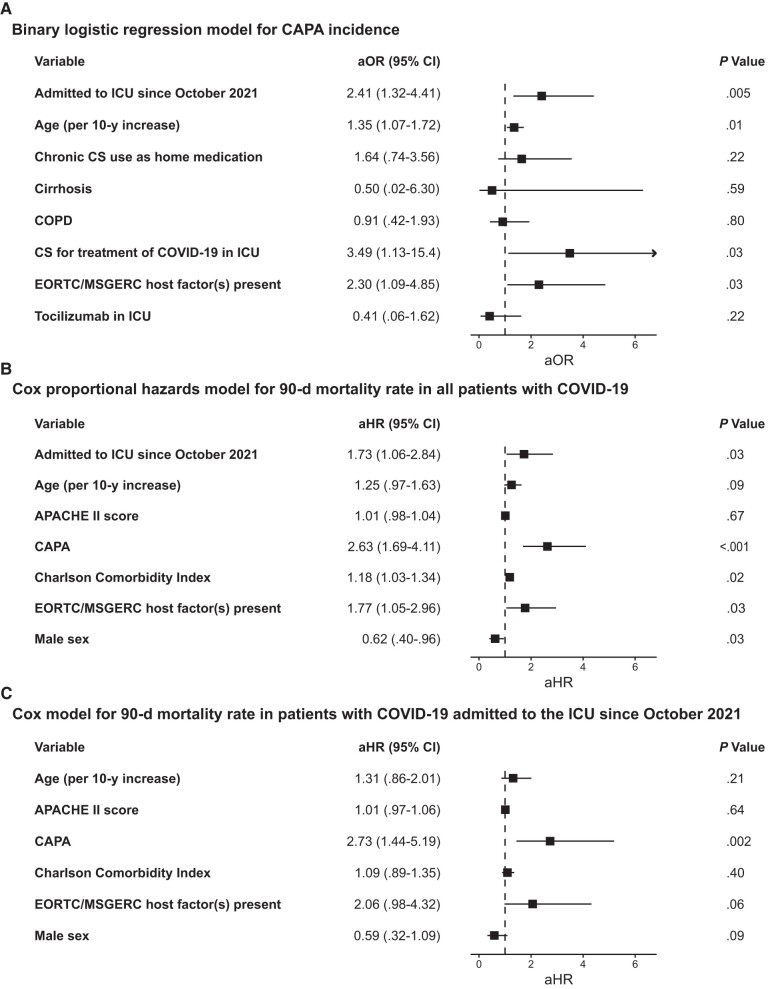
Multivariable models. *A*, Binary logistic regression model for coronavirus disease 2019 (COVID-19)–associated pulmonary aspergillosis (CAPA) incidence in mechanically ventilated patients with COVID-19 (N = 335). *B*, Cox multivariable proportional hazards model for 90-day mortality rate in patients with CAPA and those with COVID-19 only (n = 280 as 55 patients were missing Acute Physiology and Chronic Health Evaluation II [APACHE II] data). *C*, Cox multivariable proportional hazards model for 90-day mortality rate in patients with CAPA and those with COVID-19 only admitted to the intensive care unit (ICU) since October 2021 (n = 89 as 5 patients were missing APACHE II data). Note that CAPA was included as a time-dependent variable in both Cox models. Abbreviations: aHR, adjusted hazard ratio; aOR, adjusted odds ratio; COPD, chronic obstructive pulmonary disease; CS, corticosteroid; EORTC, European Organisation for Research and Treatment of Cancer; MSGERC, Mycosis Study Group Education and Research Consortium.

Patients with CAPA required more renal replacement therapy than those without CAPA (33% (37 of 112) vs 17% (38 of 223); *P* = .001), had longer ICU stays (median [IQR], 32 [21–49] vs 21 [13–33] days; *P* < .001) and hospital stays (median, 46 [30–79] vs 33 [23–53] days; *P* < .001). Patients with CAPA had a 90-day mortality rate of 48% (54 of 112 patients) after ICU admission, significantly higher than the 21% (47 of 223) observed in patients without CAPA (visualized with landmark Kaplan-Meier curve in Figure 2). We assessed whether CAPA development was independently associated with mortality rate in our cohort using Cox multivariate proportional hazards models. In the first model, we looked at the 90-day mortality rate in the total cohort, incorporating the Charlson comorbidity index, the APACHE II score, male sex, age, the presence of EORTC/MSGERC host factor(s), and admittance to the ICU since October 2021 as covariates besides CAPA development, which was included as a time-dependent covariate (Figure 3). CAPA was strongly associated with 90-day mortality rate in this model (adjusted hazard ratio, 2.63 [confidence interval, 1.69–4.11]; *P* < .001).

Aspergillosis-related characteristics for the whole CAPA cohort can be found in Table 2. Of the 112 patients with CAPA, 10 (9%) had proven disease, while the other 102 (91%) had probable aspergillosis, according to the ECMM/ISHAM criteria. Almost all patients with CAPA had a positive BAL GM result, and more than half of the patients had a positive BAL culture for *Aspergillus* species. *Aspergillus fumigatus* was by far the most encountered species (cultured in 93% [57 of 61 patients]). Overall, the prevalence of angioinvasion as reflected by a positive serum GM result was low (10 patients [9%]). The diagnosis of CAPA (defined as the day the first sample with evidence for CAPA was retrieved) was made at median (IQR) of 7 (3–13) days after ICU admission. Antifungals were administered in 99% of patients with CAPA (111 of 112; 1 patient died on the day of diagnosis). Azoles (voriconazole, isavuconazole, or posaconazole) were the preferred antifungals, and the median (IQR) duration of antifungal therapy was 24 (11–42) days in all patients with CAPA and 40 (23–60) days in survivors. Importantly, histopathological examinations of biopsy samples (tracheobronchial biopsy samples obtained in vivo or postmortem transthoracic lung biopsy samples) or full autopsies were performed in 18 patients with CAPA. Aspergillosis was proved in 10 of these patients, despite administration of antifungal therapy in all patients before biopsy or autopsy.

**Table 2.** Aspergillosis-Related Characteristics in Patients With Coronavirus Disease 2019–Associated Pulmonary Aspergillosis

Characteristic	Patients With CAPA, No. (%)^a^			*P* Value^b^
	All Patients (n = 112)	EORTC/MSGERC Host Factor Positive (n = 50)	EORTC/MSGERC Host Factor Negative (n = 62)	
Probable aspergillosis^c^	102 (91)	45 (90)	57 (92)	.75
Proven aspergillosis^c^	10 (8.9)	5 (10)	5 (8.1)	.75
Positive BAL culture for *Aspergillus*	61 (54)	28 (56)	33 (53)	.85
*Aspergillus fumigatus*	57/61 (93)	26/28 (93)	31/33 (94)	…
*Aspergillus niger*	3/61 (4.9)	3/28 (11)	0/33 (0)	…
*Aspergillus flavus*	2/61 (3.3)	1/28 (3.6)	1/33 (3.0)	…
*Aspergillus terreus*	2/61 (3.3)	1/28 (3.6)	1/33 (3.0)	…
*Aspergillus nidulans*	1/61 (1.6)	0/28 (0)	1/33 (3.0)	…
BAL GM index ≥1.0	106 (95) (n = 111)	48 (96) (n = 50)	58 (95) (n = 61)	>.99
Highest BAL GM index throughout ICU stay, median (IQR)	4.8 (2.3–5.6) (n = 111)	5.0 (2.4–5.9) (n = 50)	4.7 (2.2–5.4) (n = 61)	.25
Serum GM index >0.5	10 (12) (n = 85)	9 (23) (n = 40)	1 (2.2) (n = 45)	.005^d^
Time between ICU admission and retrieval of 1st sample with mycological evidence of CAPA, median (IQR), d	7 (3–13)	7 (2–14)	6 (4–12)	.98
Retrieval of 1st sample with mycological evidence of CAPA before intubation	17 (15)	12 (24)	5 (8.1)	.03^d^
Received antifungal therapy targeted against aspergillosis	111 (99)	49 (98)	62 (100)	.45
Duration of antifungal therapy, median (IQR), d	24 (11–42) (n = 111)	22 (11–42) (n = 49)	25 (10–42) (n = 62)	.98
Duration of antifungal therapy in survivors, median (IQR), d	40 (23–60) (n = 58)	52 (36–85) (n = 17)	40 (20–49) (n = 41)	.13
Time between 1st sample with mycological evidence of CAPA and start of antifungal therapy, median (IQR), d	2 (1–3) (n = 111)	2 (1–2) (n = 49)	2 (1–3) (n = 62)	.31
Antifungal received				
Azole^e^	108 (96)	48 (96)	60 (97)	>.99
Liposomal amphotericin B	22 (20)	10 (20)	12 (19)	>.99
Echinocandin	5 (4.5)	2 (4.0)	3 (4.8)	>.99

Abbreviations: BAL, bronchoalveolar lavage; CAPA, coronavirus disease 2019–associated pulmonary aspergillosis; EORTC, European Organisation for Research and Treatment of Cancer; GM, galactomannan; ICU, intensive care unit; IQR, interquartile range; MSGERC, Mycosis Study Group Education and Research Consortium.

^a^Data represent no. (%) of patients unless otherwise specified.

^b^ *P* values calculated for categorical variables by Fisher exact test and for continuous variables with Student *t* test or Mann-Whitney *U* test where appropriate.

^c^According to the European Confederation for Medical Mycology/International Society for Human and Animal Mycology (ECMM/ISHAM) guidelines [18].

^d^Significant at *P* < .05.

^e^Voriconazole, isavuconazole, or posaconazole.

### CAPA in the Vaccination era

The period since October 2021, the vaccination era, saw a striking increase in the percentage of patients with severe COVID-19 with EORTC/MSGERC host factors, such as presence of a solid organ transplant or hematological cancer. Before October 2021, EORTC/MSGERC host factors were present in 10% of patients (25 of 241), which increased to 60% (56 of 94) among those admitted since October 2021 (Figure 1 and Supplementary Table 1). This is in line with the generally increased proportion of patients with severe COVID-19 with some form of immunosuppression seen in other cohorts [5, 6].

Parallel to the rise in presence of EORTC/MSGERC host factors, we observed a prominent increase in CAPA incidence from 24% (57 of 241) among patients admitted before to 59% (55 of 94) among those admitted since October 2021 (*P* < .001; Figure 1 and Supplementary Tables 1 and 2). We observed this increase both in EORTC/MSGERC-negative and EORTC/MSGERC-positive patients, the latter having an incidence as high as 70% (39 of 56) since October 2021 (Figure 1 and Supplementary Table 1). A time-to-event subdistribution competing risk analysis adjusting for ventilator weaning and death before CAPA could occur confirmed the association between CAPA incidence and EORTC/MSGERC host factor positivity and ICU admission since October 2021 (Figure 1 and Supplementary Figure 2). Increased use of corticosteroids in the ICU as a treatment modality for severe COVID-19 could have contributed to the increased incidence observed in patients without host factors (100% [38 of 38] admitted since vs 88% (189 of 216) of those admitted before the vaccination era), as well as an increase in the use of fungal diagnostics: for example, 95% host factor–negative patients (36 of 38) had BAL GM testing since the vaccination era, compared with 83% (179 of 216) before. We had information on the SARS-CoV-2 strain for 68 patients admitted to the ICU since the vaccination era, but we could not identify differences in CAPA incidence when stratifying for SARS-CoV-2 variant in this group: CAPA developed in 43% (19 of 44) infected with the Delta variant and 58% (14 of 24) infected with Omicron (*P* = .65).

Binary logistic regression analysis considering relevant covariates confirmed both EORTC/MSGERC host factor positivity and admittance to the ICU during the vaccination era as independent risk factors for CAPA development, as well as the use of corticosteroids to treat COVID-19 in the ICU (Figure 3*A*).

When comparing aspergillosis-related parameters in EORTC/MSGERC host factor–positive and host factor–negative patients with CAPA, we found a significantly higher prevalence of positive serum GM results in the former. Among patients in whom serum GM testing was performed, only 1 of 45 (2.2%) host factor–negative patients with CAPA had a positive serum GM result during ICU stay, compared with 9 of 40 (23%) host factor–positive patients (*P* = .005; Table 2). Moreover, EORTC/MSGERC host factor–positive patients had CAPA diagnosed before intubation significantly more often than host factor–negative patients. This was likely caused by a slower disease progression and thus protracted course of noninvasive ventilation before intubation in host factor–positive patients. Indeed, intubation was performed a median (IQR) of 1 (0–3) days after ICU admission in host factor–negative patients and 4 (1–8) days in host factor–positive patients (*P* < .001), while the median day of CAPA diagnosis after ICU admission did not differ significantly between the 2 groups (Table 2). Moreover, the higher likelihood of a positive serum GM result in host factor–positive patients could have aided in diagnosing CAPA before intubation. Importantly, in 5 of 6 host factor–positive patients with CAPA with in vivo or postmortem histopathological examination, invasive aspergillosis was proved (Table 2).

To evaluate whether the independent association between CAPA and 90-day mortality rate persisted in the vaccination era, we performed a multivariable Cox analysis in the patients with COVID-19 admitted to the ICU since October 2021, using the same parameters described above (Figure 3*C*). Here, development of CAPA was the only factor that was associated independently with 90-day mortality rate (adjusted hazard ratio, 2.73 [95% confidence interval, 1.44–5.19]; *P* = .002). Among all patients with CAPA, using a similar Cox model, the 90-day mortality rate was independently associated with presence of EORTC/MSGERC host factors and female sex but not with ICU admission in the vaccination era (Supplementary Figure 3).

## DISCUSSION

To our knowledge, we present the first study investigating the incidence and impact of CAPA in a large cohort of mechanically ventilated patients with COVID-19 admitted since the implementation of widespread vaccination. Owing to the inclusion of 335 patients, we were able to assess risk factors for CAPA incidence and mortality rate, such as EORTC/MSGERC host factors, in several multivariable analyses.

We observed a 33% incidence of CAPA during ICU stays (112 of 335 patients). This incidence is higher than what is reported in most observational studies on CAPA published to date. Indeed, in studies with ≥300 inclusions that used the ECMM/ISHAM definitions, ICU probable or proven CAPA incidences range between 1.1% and 20% [8–10, 17, 21–29]. This wide variety in reported CAPA incidences has been attributed to several causes, such as pure geographic variability, variable numbers of patients who received adequate fungal workup, variation in the use of GM testing as a diagnostic tool, and prospective or retrospective study design [7, 30].

Our thorough fungal workup (with 90% of our patients undergoing bronchoscopy with BAL sampling, mostly combined with culture and GM testing) and the fact that we included only mechanically ventilated patients contribute to the high CAPA incidence reported in our study. However, the main reason appears to be the inclusion of patients during the vaccination era. The aforementioned studies were all (largely) conducted in patients admitted to ICU before vaccination programs had been rolled out, and the patient profiles included in those studies therefore differ significantly from the profiles that are nowadays considered at risk for respiratory failure requiring ICU admission due to severe COVID-19.

In our study, we observed that fewer patients required mechanical ventilation for COVID-19 since widespread implementation of vaccination, but those who did require mechanical ventilation were more likely to present an EORTC/MSGERC host factor for invasive mold disease (10% before vs 60% since the vaccination era). Consequently, we found that the incidence of ECMM/ISHAM probable and proven CAPA increased from 24% in patients admitted to ICU before to 59% in those admitted since the vaccination era. Importantly, 70% of mechanically ventilated EORTC/MSGERC host factor–positive patients admitted to our ICU since October 2021 developed CAPA. Moreover, in patients without preexisting host factors, the incidence of CAPA increased ever since the vaccination era, most likely owing to a combination of increased awareness and thus increased detection and to a higher proportion of patients receiving corticosteroids to treat severe COVID-19. In our center, only tocilizumab was used as an additional immunomodulatory therapy for severe COVID-19 in a limited number of cases, and its use was not significantly higher in the vaccination era. We found no arguments for an increased CAPA incidence in COVID-19 due to Omicron compared with Delta SARS-CoV-2 variants.

We corroborated the clear association between CAPA and mortality rate observed in prior studies [9, 17]. Moreover, we showed that this association persists in the vaccination era. To date, only small observational studies have described the effect of antifungal prophylaxis in patients with severe COVID-19 [31–34]. Each of these studies showed a benefit of inhaled or systemic prophylaxis on the incidence of CAPA. Given our observation that CAPA incidence and mortality rate, respectively, increased and persisted in the vaccination era, our results call for randomized, controlled trials assessing the potential of antifungal prophylaxis in patients with severe COVID-19.

Our study has several limitations. First, we conducted a retrospective monocentric study, and therefore our findings call for an international multicenter (and preferably prospective) trial to confirm that this association is present in other centers and countries as well. Second, our center is a tertiary referral and large transplantation center. Consequently, the incidences observed here are likely to be lower in centers without transplantation activities or with a low rate of severely immunocompromised patients among their patients. Third, we chose to divide the cohorts according to their admission date in regard to a “vaccination era” with high vaccination coverage in the general population, rather than by individual vaccination status. Our approach accounted for herd immunity but forsakes the individual variation in vaccination status between patients.

Fourth, and probably the most important limitation, is the fact that the criteria to diagnose probable aspergillosis in patients with severe COVID-19, and by extension all critically ill patients, have not been validated extensively against the gold standard of histology. As this is a general problem for all invasive fungal disease classification criteria available, this issue is difficult to overcome, given the high risk associated with obtaining biopsy material from severely diseased lungs and the selection bias associated with reliance on autopsies only. The consequence is that the current criteria probably lead to a certain degree of misidentification of very early *Aspergillus* infection/colonization stages as invasive disease [35]. Few autopsy studies have been performed, some (including a large systematic review) showing a much lower incidence of proven CAPA compared with the relatively high incidences of probable CAPA in many observational studies [36, 37]. However, sampling bias and lack of use of stains specific for fungal disease might lead to underestimation of proven CAPA.

With this in mind, a recent study by our group showed that in half of patients with severe COVID-19 who died and received an autopsy and had a diagnosis of probable CAPA during life, invasive aspergillosis was still found, even after a median antifungal therapy duration of 9 days [38]. Similar findings were observed in the autopsy studies by Fortarezza et al [39] and Casalini et al [40] and were corroborated in our study population as well. Nevertheless, despite this uncertain degree of colonization in patients categorized as having probable CAPA by the current ECMM/ISHAM criteria, the increase in incidence in our center remains a relevant finding and calls for further investigation.

In conclusion, we show that the incidence of CAPA in mechanically ventilated patients with COVID-19 increased significantly since the widespread implementation of vaccination against SARS-CoV-2. This increase can be mainly attributed to an increase in the fraction of patients with EORTC/MSGERC host factors for invasive mold disease. Our findings warrant setting up new prospective, international observational studies for CAPA in the vaccination era, and they show the urgency for investigating antifungal prophylaxis in critically ill patients with COVID-19.

## Supplementary Data

Supplementary materials are available at *Clinical Infectious Diseases* online. Consisting of data provided by the authors to benefit the reader, the posted materials are not copyedited and are the sole responsibility of the authors, so questions or comments should be addressed to the corresponding author.

## Supplementary Material

ciad546_Supplementary_Data

## Notes

***Author Contributions.*** Conceptualization: S. F., K. L., M. H., and J. W. Methodology: S. F., M. B., and J. W. Investigation: S. F., K. L., H. M. L., K. H., C. J., Y. D., G. H., J. M., P. M., M. P., I. S., C. V., L. V., R. V., E. V. W., A. W., and J. W. Formal analysis: S. F. and M. B. Visualization: S. F. Writing—original draft: S. F. and J. W. Writing—review and editing: All authors.

***Financial support.*** This work was supported by internal funding by KU Leuven and University Hospitals Leuven (support to J. W.) and the Research Foundation–Flanders (Fonds Wetenschappelijk Onderzoek, FWO; PhD fellowship 11M6922N to S. F).
