# Supplementary material for: High Burden of COVID-19-Associated Pulmonary Aspergillosis in Severely Immunocompromised Patients Requiring Mechanical Ventilation
Source: Clin Infect Dis. 2023 Sep 11;78(2):361–70. doi: 10.1093/cid/ciad546 (PMC10874259; doi:10.1093/cid/ciad546)
Supplement: ciad546_Supplementary_Data [file ciad546_supplementary_data.docx]

**Supplementary information to:**

**High burden of COVID-19-associated pulmonary aspergillosis (CAPA) in severely immunocompromised patients requiring mechanical ventilation**

Simon Feys, Katrien Lagrou, Hanne Moon Lauwers, Koen Haenen, Cato Jacobs, Marius Brusselmans, Yves Debaveye, Greet Hermans, Martin Hoenigl, Johan Maertens, Philippe Meersseman, Marijke Peetermans, Isabel Spriet, Christophe Vandenbriele, Lore Vanderbeke, Robin Vos, Eric Van Wijngaerden, Alexander Wilmer, Joost Wauters

**Supplementary Table 1. Characteristics of patients requiring mechanical ventilation for COVID-19 before vs since October 2021.**

|  | | | | Before Oct 2021  n = 241 | After Oct 2021  n = 94 | *P-value* |
| --- | --- | --- | --- | --- | --- | --- |
| Male sex | | | | 171 (71%) | 67 (71%) | 1 |
| Mean age – years (SD) | | | | 63 (12) | 64 (10) | 0.58 |
| Median BMI (IQR) | | | | 29 (26-33) | 26 (23-29) | **<0.0001** |
| Completed vaccination against SARS-CoV-2^†^ | | | | 13 (5.4%) | 73 (78%) | **<0.0001** |
| COPD | | | | 25 (10%) | 17 (18%) | 0.066 |
| Liver cirrhosis | | | | 2 (0.83%) | 1 (1.1%) | 1 |
| Diabetes mellitus | | | | 94 (39%) | 28 (30%) | 0.13 |
| EORTC/MSGERC host factor* | | | | 25 (10%) | 56 (60%) | **<0.0001** |
|  | | Solid organ transplant | | 14 (5.8%) | 36 (38%) | **<0.0001** |
|  | | | Lung transplant | 5 (2.1%) | 24 (26%) | **<0.0001** |
|  | | | Kidney transplant | 4 (1.7%) | 10 (11%) | **0.00072** |
|  | | | Liver transplant | 2 (0.83%) | 1 (1.1%) | 1 |
|  | | | Heart transplant | 3 (1.2%) | 2 (2.1%) | 0.62 |
|  | | Hematological malignancy | | 7 (2.9%) | 14 (15%) | **0.00016** |
|  | | Allogeneic HSCT | | 1 (0.41%) | 4 (4.3%) | **0.023** |
|  | | Acute GVHD grade III-IV involving gut, lungs or liver refractory to first-line CS | | 0 (0%) | 2 (2.1%) | 0.078 |
|  | | Recent prolonged neutropenia | | 1 (0.41%) | 0 (0%) | 1 |
|  | | Recent prolonged high-dose CS | | 1 (0.41%) | 5 (5.3%) | **0.0075** |
|  | | T or B cell immunosuppressants | | 21 (8.7%) | 50 (53%) | **<0.0001** |
|  | | Inherited severe immunodeficiency | | 0 (0%) | 0 (0%) |  |
| Low-dose CS^§^ as home medication | | | | 26 (11%) | 43 (46%) | **<0.0001** |
| Median APACHE II score at ICU admission (IQR) | | | | 19 (16-27)  n = 191 | 18 (15-24)  n = 89 | 0.060 |
| Median Charlson Comorbidity Index at ICU admission (IQR) | | | | 3 (2-5) | 3 (2-4) | 0.48 |
| CS (daily dose ≥20 mg prednisone equivalent) as treatment for COVID-19 during ICU stay | | | | 212 (88%) | 94 (100%) | **<0.0001** |
| Tocilizumab during hospital stay | | | | 14 (5.8%) | 2 (2.1%) | 0.25 |
| Received MV | | | | 241 (100%) | 94 (100%) | 1 |
|  | | Median days of MV (IQR) | | 14 (8-22) | 17 (9-35) | **0.030** |
| Received ECMO | | | | 52 (22%) | 16 (17%) | 0.45 |
|  | | Median days of ECMO (IQR) | | 13 (9-20)  n = 52 | 21 (14-29)  n = 16 | **0.044** |
| Required renal replacement therapy | | | | 45 (19%) | 30 (32%) | **0.013** |
| Median days ICU stay (IQR) | | | | 22 (14-34) | 33 (17-49) | **0.0011** |
| Median days hospital stay (IQR) | | | | 34 (24-53) | 45 (24-79) | **0.0085** |
| 90-day mortality after ICU admission | | | | 58 (24%) | 43 (46%) | **0.00018** |
| At least one BAL sampling performed | | | | 209 (87%) | 91 (97%) | **0.0051** |
| At least one BAL GM tested | | | | 199 (83%) | 91 (97%) | **0.00028** |
| At least one serum GM tested | | | | 127 (53%) | 61 (65%) | 0.050 |
| Developed CAPA during ICU stay | | | | 57 (24%) | 55 (59%) | **<0.0001** |
|  | CAPA in EORTC/MSGERC host factor negative patients | | | 46/216 (21%) | 16/38 (42%) | **0.013** |
|  | CAPA in EORTC/MSGERC host factor positive patients | | | 11/25 (44%) | 39/56 (70%) | **0.047** |

P-values calculated for categorical variables by Fisher’s exact test and for continuous variables with Student’s *t*-test or Mann-Whitney *U*-test where appropriate. APACHE II: Acute Physiology and Chronic Health Evaluation II; BAL: bronchoalveolar lavage; BMI: body mass index; COPD: chronic obstructive pulmonary disease; CS: corticosteroids; ECMO: extracorporeal membrane oxygenation; EORTC: European Organisation for Research and Treatment of Cancer; GM: galactomannan; GVHD: graft vs host disease; ICU: intensive care unit; IQR: interquartile range; MV: mechanical ventilation; Mycosis Study Group Education and Research Consortium. ^†^ Having received two COVID-19 vaccination doses (mRNA-based BNT162b2 (Comirnaty) or mRNA-1273 (Spikevax), or viral vector-based AZD1222 (Vaxzevria)) or one dose of viral vector-based Ad26.COV2.S (Jcovden) prior to hospitalization. * EORTC/MSGERC host factors for invasive mold disease [1]. ^§^ Daily dose below the EORTC/MSGERC corticosteroid host factor cut-off as chronic home medication.

**Supplementary Table 2. Aspergillosis-related characteristics in CAPA patients admitted before vs since October 2021.**

|  | | CAPA & admitted before Oct 2021  n = 57 | CAPA & admitted since Oct 2021  n = 55 | p*-*value |
| --- | --- | --- | --- | --- |
| Probable aspergillosis* | | 51 (89%) | 51 (93%) | 0.74 |
| Proven aspergillosis* | | 6 (11%) | 4 (7.3%) | 0.74 |
| Positive BAL culture for *Aspergillus* | | 28 (49%) | 33 (60%) | 0.26 |
|  | *Aspergillus fumigatus* | 25/28 (89%) | 32 (97%) |  |
|  | *Aspergillus niger* | 2/28 (7.1%) | 1/33 (3.0%) |  |
|  | *Aspergillus flavus* | 1/28 (3.6%) | 1/33 (3.0%) |  |
|  | *Aspergillus terreus* | 1/28 (3.6%) | 1/33 (3.0%) |  |
|  | *Aspergillus nidulans* | 1/28 (3.6%) | 0/33 (0%) |  |
| BAL GM ≥ 1.0 | | 55 (96%) n = 56 | 51 (93%) n = 55 | 0.21 |
|  | Median highest BAL GM value throughout ICU stay | 4.6 (2.7-5.6) | 4.9 (2.0-5.6) | 0.88 |
| Serum GM > 0.5 | | 2 (3.5%)  n = 43 | 8 (15%)  n = 42 | **0.049** |
| Median days between ICU admission and retrieval first sample with mycological evidence for CAPA | | 6 (3-12) | 7 (3-14) | 0.62 |
| First sample with mycological evidence for CAPA retrieved before intubation | | 3 (5.3%) | 14 (25%) | **0.0034** |
| Received antifungal therapy targeted against aspergillosis | | 57 (100%) | 54 (98%) | 0.49 |
|  | Median days of antifungal therapy (IQR) | 25 (10-42) | 24 (12-44) | 0.61 |
|  | Median days of antifungal therapy in survivors (IQR) | 37 (20-50)  n = 34 | 47 (29-73)  n = 24 | 0.13 |
|  | Median days between first sample with mycological evidence for CAPA and start antifungal therapy | 2 (1-2) | 2 (1-3) | 0.58 |
|  | Azole^§^ | 55 (96%) | 53 (96%) | 1 |
|  | Liposomal amphotericin B | 13 (23%) | 9 (16%) | 0.48 |
|  | Echinocandin | 3 (5.3%) | 2 (3.6%) | 1 |

P-values calculated for categorical variables by Fisher’s exact test and for continuous variables with Student’s *t*-test or Mann-Whitney *U*-test where appropriate. CAPA: COVID-19-associated pulmonary aspergillosis; BAL: bronchoalveolar lavage; GM: galactomannan.
* According to the ECMM/ISHAM guidelines [2]; § Voriconazole, isavuconazole or posaconazole

**Supplementary Figure 1. Inclusions and exclusions diagram.**

**
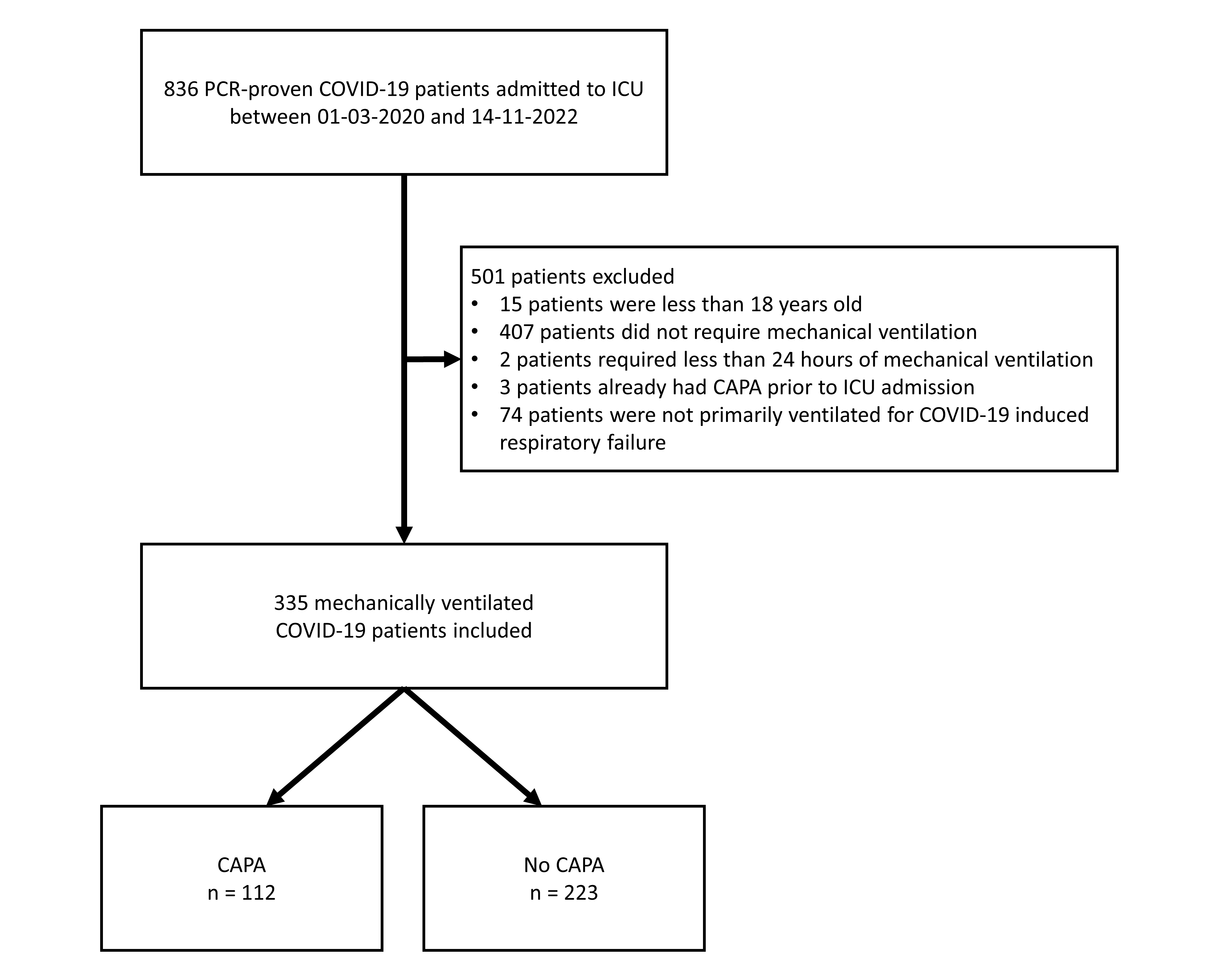
**

**Supplementary Figure 2. Fine and Gray model.**

**
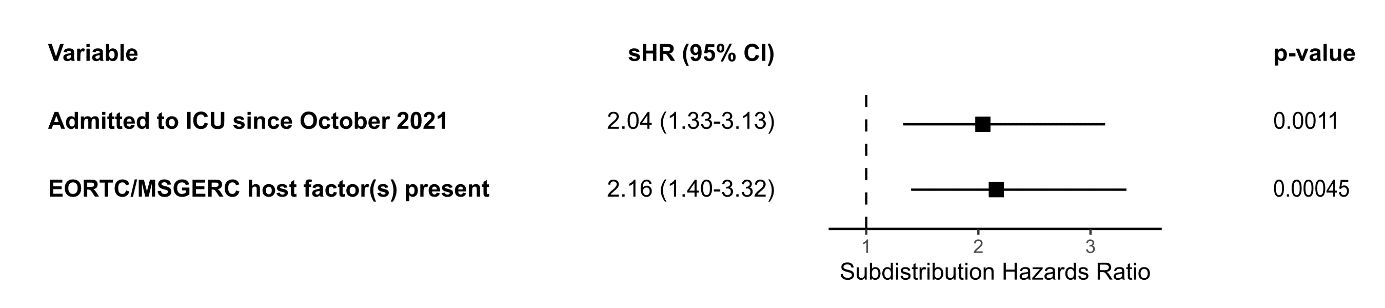
**

Fine and Gray model for 45-day CAPA incidence correcting for competing risks (extubation or death).
sHR: subdistribution Hazard Ratio; EORTC: European Organisation for Research and Treatment of Cancer; ICU: intensive care unit; MSGERC: Mycosis Study Group Education and Research Consortium.

**Supplementary Figure 3. Cox proportional hazards model for mortality in all CAPA patients**

**
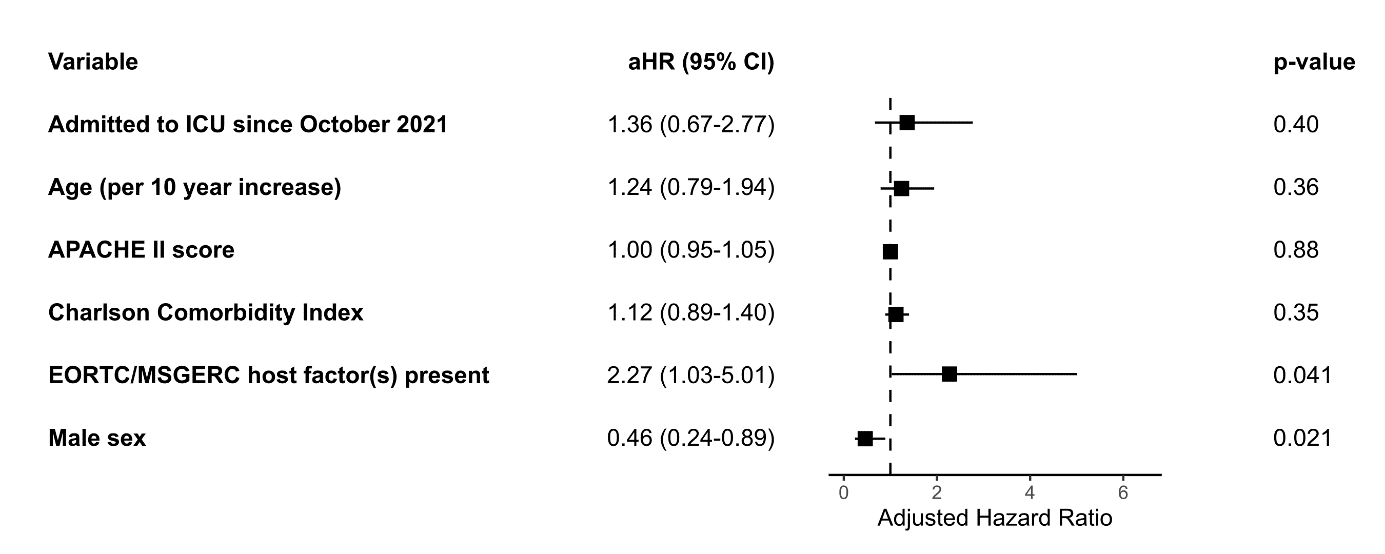
**

Cox multivariable proportional hazards model for mortality in CAPA patients, starting from CAPA diagnosis (censoring at 90 days after ICU admission). N = 98 due to 14 patients with missing APACHE II data.
aHR: adjusted Hazard Ratio; APACHE II: Acute Physiology and Chronic Health Evaluation II; EORTC: European Organisation for Research and Treatment of Cancer; ICU: intensive care unit; MSGERC: Mycosis Study Group Education and Research Consortium.

**References**

1. Donnelly JP, Chen SC, Kauffman CA, et al. Revision and Update of the Consensus Definitions of Invasive Fungal Disease From the European Organization for Research and Treatment of Cancer and the Mycoses Study Group Education and Research Consortium. Clinical Infectious Diseases **2020**; 71:1367–1376.

2. Koehler P, Bassetti M, Chakrabarti A, et al. Defining and managing COVID-19-associated pulmonary aspergillosis: the 2020 ECMM/ISHAM consensus criteria for research and clinical guidance. The Lancet Infectious Diseases **2021**; 21:e149–e162.
